# Surface Immobilization of Nano-Silver on Polymeric Medical Devices to Prevent Bacterial Biofilm Formation

**DOI:** 10.3390/pathogens8030093

**Published:** 2019-06-28

**Authors:** Andri K. Riau, Thet T. Aung, Melina Setiawan, Liang Yang, Gary H. F. Yam, Roger W. Beuerman, Subbu S. Venkatraman, Jodhbir S. Mehta

**Affiliations:** 1 Tissue Engineering and Stem Cell Group, Singapore Eye Research Institute, Singapore 169856, Singapore; 2 School of Materials Science and Engineering, Nanyang Technological University, Singapore 639798, Singapore; 3 Anti-Infectives Research Group, Singapore Eye Research Institute, Singapore 169856, Singapore; 4 School of Medicine, Southern University of Science and Technology, Shenzhen 518055, China; 5 Ophthalmology and Visual Sciences ACP, Duke-National University of Singapore Medical School, Singapore 169857, Singapore; 6 SRP Neuroscience and Emerging Infectious Disease, Duke-National University of Singapore Medical School, Singapore 169857, Singapore; 7 Corneal and External Eye Disease Service, Singapore National Eye Centre, Singapore 168751, Singapore

**Keywords:** silver, toxicity, cornea, biofilm, polymer, nanoparticles

## Abstract

Bacterial biofilm on medical devices is difficult to eradicate. Many have capitalized the anti-infective capability of silver ions (Ag^+^) by incorporating nano-silver (nAg) in a biodegradable coating, which is then laid on polymeric medical devices. However, such coating can be subjected to premature dissolution, particularly in harsh diseased tissue microenvironment, leading to rapid nAg clearance. It stands to reason that impregnating nAg directly onto the device, at the surface, is a more ideal solution. We tested this concept for a corneal prosthesis by immobilizing nAg and nano-hydroxyapatite (nHAp) on poly(methyl methacrylate), and tested its biocompatibility with human stromal cells and antimicrobial performance against biofilm-forming pathogens, *Pseudomonas aeruginosa* and *Staphylococcus aureus*. Three different dual-functionalized substrates—high Ag (referred to as 75:25 HAp:Ag); intermediate Ag (95:5 HAp:Ag); and low Ag (99:1 HAp:Ag) were studied. The 75:25 HAp:Ag was effective in inhibiting biofilm formation, but was cytotoxic. The 95:5 HAp:Ag showed the best selectivity among the three substrates; it prevented biofilm formation of both pathogens and had excellent biocompatibility. The coating was also effective in eliminating non-adherent bacteria in the culture media. However, a 28-day incubation in artificial tear fluid revealed a ~40% reduction in Ag^+^ release, compared to freshly-coated substrates. The reduction affected the inhibition of *S. aureus* growth, but not the *P. aeruginosa*. Our findings suggest that Ag^+^ released from surface-immobilized nAg diminishes over time and becomes less effective in suppressing biofilm formation of Gram-positive bacteria, such as *S. aureus*. This advocates the coating, more as a protection against perioperative and early postoperative infections, and less as a long-term preventive solution.

## 1. Introduction

Biofilm formation is initiated when planktonic bacterial cells attach on, multiply, and form a community of microorganisms on living and non-living surfaces, e.g., on the surface of water pipes, rocks, teeth, cornea, and implanted medical devices [1]. Medical devices are particularly susceptible to bacterial adherence and biofilm formation, especially those that extend from a sterile environment (e.g., blood or anterior chamber) to the surface of the body (e.g., skin or ocular surface), such as cardiac ventricular assist devices or corneal prosthetic devices (KPros). Patients treated with this type of medical devices require life-long antibiotic treatment, subjecting them to the risk of developing antibiotic-resistant infections [2]. An increasing number of antibiotic-resistant infection cases have been associated with microorganisms protected in biofilms on indwelling devices [3,4]. Biofilms are reported to be up to a 1,000-fold more resistant to antibiotics, compared to planktonic bacteria, because of poor drug or macromolecule penetration into the biofilm’s extracellular matrix and the adaptive bacterial response to environmental stress [5]. In these situations, the infected device has a high tendency to fail and eventually requires removal and replacement in order to control and eradicate the infection.

Among metal nanoparticles with proven antimicrobial activity, silver nanoparticles (nAg) are an especially potent and effective bactericidal agent [6]. Currently, the use of nAg can be found in wound care products, textiles, cosmetics, and household antiseptic sprays [7,8]. It has also been proposed as a candidate material for coating medical devices [7,9,10]. Silver is typically inert, but in the presence of tissues, when implanted, becomes ionized due to the presence of oxygen, moisture, and body fluids, releasing biologically active silver ions (Ag^+^) that bind to thiol groups and anionic ligands of proteins and cell membranes of bacterial cells [11,12]. The basis of the antimicrobial action of silver is attributed to the ability of Ag^+^ to penetrate bacterial cell walls via pinocytosis, causing denaturation and inactivation of proteins and also metabolic enzymes that lead to growth-arrest in the bacteria [11,12,13].

**Figure 1:**
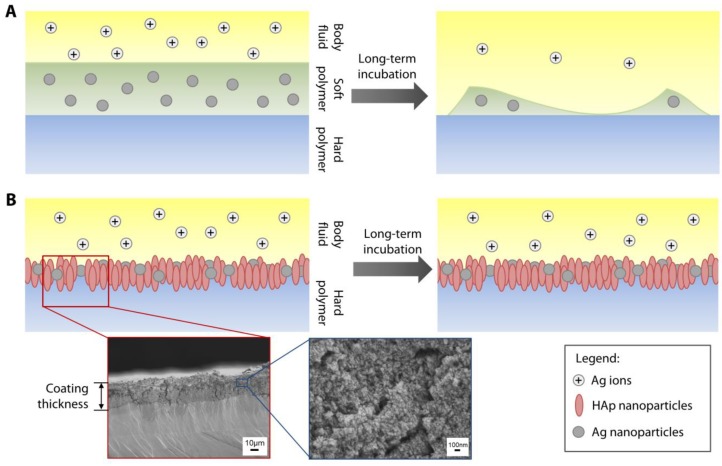
Illustration of methods to functionalize hard polymer with antimicrobial silver nanoparticles. (**A**) Most studies in the literature describe incorporation of silver nanoparticles (nAg) or salts into a soft and biodegradable polymeric coating that is laid on a hard polymer or substrate. (**B**) Our method involves direct immobilization of the nAg on the surface of the hard polymer. The antimicrobial function of Ag can be coupled with cell support function provided by hydroxyapatite (HAp) NPs to create dual functionalization of the substrate. Since there is no biodegradable component needed in our proposed coating technique, the nAg can be retained as long the hard polymer is not degraded; hence, the Ag ions, released by the nAg, could be available locally for a significantly longer time. Cross-sectional SEM of an actual polymethylmethacrylate (PMMA) sheet coated with nAg and nHAp. The thickness of the coating was measured at 32 ± 3 µm. Individual nanoparticles could be discerned at 30,000× magnification, as shown in the SEM image with a blue border.

Previously, researchers have capitalized on the intrinsic antimicrobial activity of Ag^+^ by incorporating nAg or salts into polymeric coatings, that are then laid on the surface of hard substrates or medical devices (Figure 1A) [14,15,16,17]. The majority of the proposed polymeric coatings are typically composed of soft polymers that are porous and are highly biodegradable, e.g., polyamide, chitosan, or poly(N-isopropylacrylamide) (pNIPAm), to allow free movement of Ag^+^ [14,15,16,17]. However, in some clinical applications, where the microenvironment around the medical devices is highly acidic due to inflammation, soft polymers degrade prematurely and hence, a long-term availability of the Ag^+^, released from the encapsulated nAg, might not be achievable due to the rapid clearance of the free nanoparticles (NPs) from the site (Figure 1A). In addition, acute exposure to a high concentration of nAg due to the rapid degradation of the encapsulating polymer matrix could be toxic to the host cells [18,19]. Additionally, the rapid degradation of the coating would not allow sufficient time for host tissue integration into the device and would weaken the linkage between the tissue and polymethylmethacrylate (PMMA). Hence, direct attachment between the tissue and a hard substrate is required, such as that in a KPro (where corneal stromal tissue is attached around an optic cylinder made of polymethylmethacrylate (PMMA) [20,21]. 

**Figure 2:**
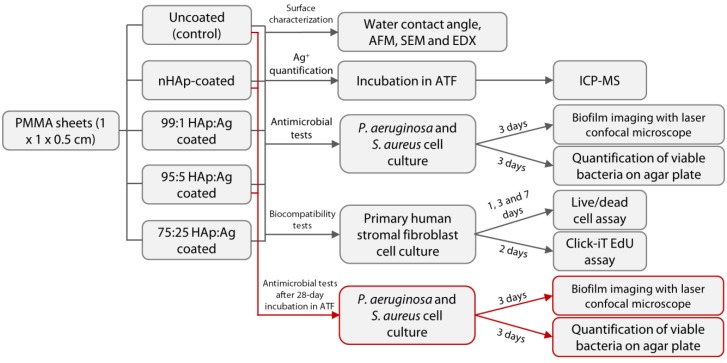
Schematic diagram of the overall experimental setup of the study. Abbreviations: nHAp = nano-hydroxyapatite; Ag = silver; PMMA = poly(methyl methacrylate); AFM = atomic force microscopy; SEM = scanning electron microscopy; EDX = energy dispersive X-ray spectroscopy; ATF = artificial tear fluid; and ICP-MS = inductively-coupled plasma-mass spectrometry.

We have previously shown that bioceramic-based NPs, such as nano-hydroxyapatite (nHAp), could be directly impregnated on the surface of PMMA and that the presence of nHAp improved the biocompatibility of PMMA [22]. As an extension of this application, herein, we immobilized nAg on the surface of nHAp-coated PMMA to create a dual functionality (antimicrobial and host cell support) to a hard substrate (Figure 1B). As summarized in the workflow diagram in Figure 2, the antimicrobial functionality of the coating was tested against *Staphylococcus aureus* and *Pseudomonas aeruginosa*, two of the most common biofilm-forming pathogens in the cornea [23,24]. The biocompatibility of corneal stromal fibroblasts in terms of cell viability, adhesion, and proliferation was also assessed, to study the potential cytotoxicity of the coating. Finally, the longer-term antimicrobial function of the coating, following a 28-day incubation in an aqueous environment was investigated.

## 2. Results

### 2.1. Surface Characterization of Dual-functionalized PMMA Surfaces

**Figure 3:**
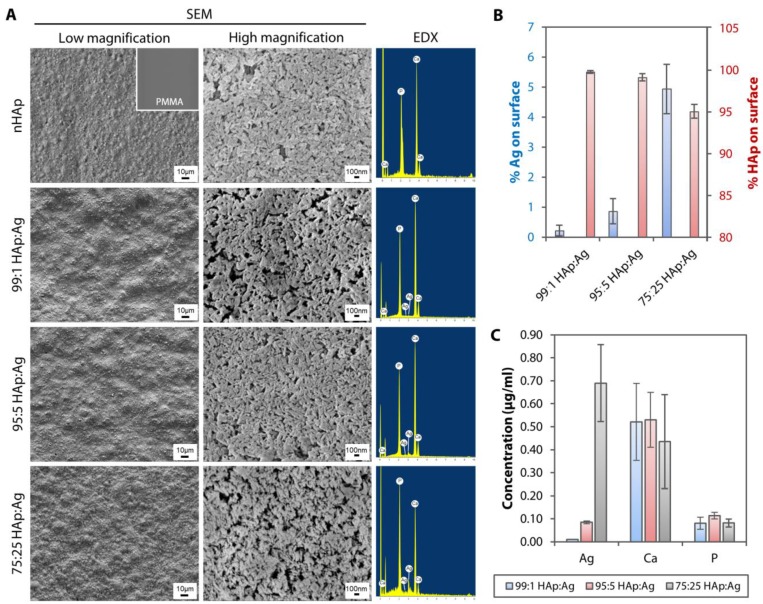
Surface morphology, surface elemental distribution, and ionic leaching of hydroxyapatite and silver-coated PMMA sheets. (**A**) SEM images under low magnification (500×) showed undulations and rough texture on HAp and Ag nanoparticle-coated PMMA surfaces. Individual nHAp, but not nAg, were distinguishable under high magnification (30,000×) on the coated PMMA surfaces. EDX confirmed the presence of Ag element on the dual-functionalized PMMA substrates. (**B**) The Ag composition was commensurate with the amount of nAg added in the dipcoating mixture. The bar graph summarizes the percentage composition of Ag and HAp (Ca and P elements combined) on the dual-functionalized PMMA. (**C**) Following a day incubation in distilled water, ICP-MS detected Ag ions that had leached from the HAp and Ag-coated PMMA sheets. The Ag concentration was commensurate with the amount of nAg added in the dipcoating mixture. The bar graph shows the concentration of trace elements, Ag, Ca, and P, detected by ICP-MS from the three coated substrates. In both bar graphs, the height of bars and height of error bars represent the mean value and standard deviation of the mean, respectively.

**Figure 4:**
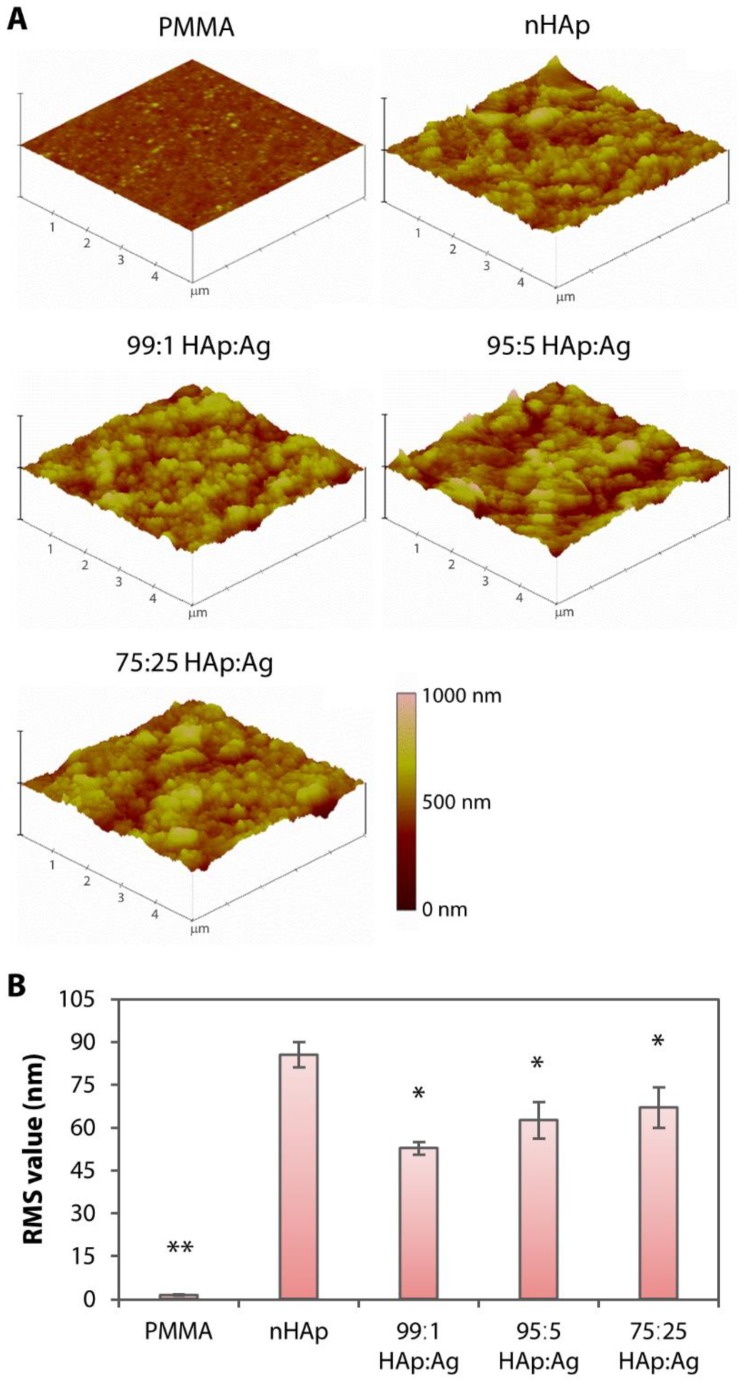
Surface roughness of hydroxyapatite and silver-coated PMMA sheets. (**A**) Three-dimensional rendering of surface topography generated by AFM. Untreated PMMA appeared relatively smooth. In contrast, nHAp or nAg-immobilized PMMA surfaces appeared significantly rougher than the pristine PMMA surface. (**B**) Bar graph summarizing the roughness or RMS value of the non-coated and coated PMMA surfaces. Height of bars and height of error bars represent average RMS value and the standard deviation of the mean, respectively. * *p* < 0.05 and ** *p* < 0.001 relative to the nHAp group.

The untreated PMMA surface was smooth and appeared featureless (see inset on the upper left most scanning electron microscopy (SEM) image in Figure 3A) with a RMS value of 1.5 ± 0.3 nm (Figure 4A). In contrast, it was obvious that the PMMA surfaces coated with nHAp or nAg appeared rougher (Figure 3A and Figure 4A). The nHAp surface was the roughest with a root mean square (RMS) value of 85.6 ± 4.4 nm and was significantly rougher than the surface of 99:1 HAp:Ag (52.7 ± 2.3 nm; *p* = 0.001), 95:5 HAp:Ag (62.6 ± 6.5 nm; *p* = 0.010), and 75:25 HAp:Ag (67.0 ± 7.2 nm; *p* = 0.026) (Figure 4B). From SEM images under high magnification, individual rod-shaped nHAp were easily distinguished (Figure 3A). On the other hand, the spherical nAg were difficult to ascertain even on the 75:25 HAp:Ag surface. However, energy dispersive X-ray (EDX) detected the presence of Ag that was commensurate with the amount of nAg added into the dipcoating mixture (Figure 3A). There was 0.22 ± 0.18%, 0.86 ± 0.42%, and 4.94 ± 0.82% Ag element (the rest of the coating components was HAp) distributed on the surface of 99:1 HAp:Ag, 95:5 HAp:Ag, and 75:25 HAp:Ag substrates, respectively (Figure 3B). We did not find any Ag element present on the nHAp surface.

In terms of hydrophilicity, all coated samples had significantly lower water contact angle (WCA), compared to the untreated PMMA (Table 1). There was a trend towards increasing hydrophobicity with an increasing amount of Ag in the coating components, but we did not find any statistical significance between the groups with Ag addition and the group with nHAp only.

Using inductively coupled plasma-mass spectrometry (ICP-MS), we detected Ag^+^ leaching into the aqueous environment after 1-day incubation. The concentration of Ag^+^ was commensurate with the amount of nAg added into the mixture (Figure 3C). We found 0.010 ± 0.001 µg/mL, 0.085 ± 0.006 µg/mL, and 0.690 ± 0.167 µg/mL of Ag^+^ from 99:1 HAp:Ag, 95:5 HAp:Ag, and 75:25 HAp:Ag substrates, respectively. From the bar graph, we could see that the calcium (Ca) and phosphorus (P) elements that had leached from the three substrates, did not differ significantly from each other (Figure 3C).

### 2.2. Prevention of Biofilm Formation by Dual-functionalized PMMA Substrates

**Figure 5:**
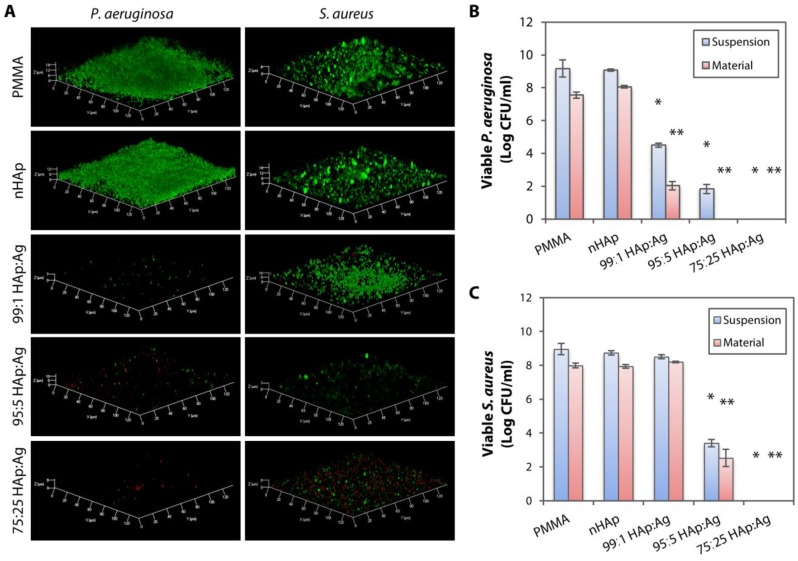
Bacterial biofilm imaging and quantification of bacterial colony forming units on hydroxyapatite and silver-coated PMMA sheets. (**A**) Laser confocal microscopy revealed *P. aeruginosa* and *S. aureus* biofilm formation on uncoated PMMA and nHAp-coated PMMA. The addition of Ag nanoparticles into nHAp substrates was more effective in inhibiting *P. aeruginosa* biofilm formation than in preventing *S. aureus* growth. (**B**) Bacterial quantification on tryptic soy agar plates confirmed the confocal imaging results, showing efficient growth inhibition (>99% killing efficiency compared to PMMA and nHAp groups) of both non-adherent *P. aeruginosa* (labelled as suspension) and *P. aeruginosa* that was still attached on the substrates (labelled as material) with all three dual-functionalized PMMA sheets. The bar graph summarizes the viable colony forming unit (CFU) of *P. aeruginosa* in the presence of untreated, nHAp-coated, and nHAp- and Ag-coated PMMA substrates. (**C**) Only 95:5 HAp:Ag and 75:25 HAp:Ag were able to inhibit the non-adherent and adherent *S. aureus*. The bar graph summarizes the viable CFU of *S. aureus* in the presence of untreated, nHAp-coated, and nHAp- and Ag-coated PMMA substrates. Height of bars and height of error bars represent mean log bacterial CFU and the standard deviation of the mean, respectively. * *p* < 0.05 and ** *p* < 0.05 relative to viable bacteria in suspension and attached on material, respectively, in the nHAp group.

Without Ag, both the pristine PMMA and nHAp substrate were unable to prevent the *P. aeruginosa* and *S. aureus* biofilm formation (Figure 5A). The addition of Ag into nHAp substrates was more effective in inhibiting the *P. aeruginosa* biofilm formation than in preventing *S. aureus* growth. There was barely any *P. aeruginosa* cells growing, even on 99:1 HAp:Ag. In contrast, *S. aureus* biofilm was clearly visible on the 99:1 HAp:Ag substrate. Reduced viable *S. aureus* was seen on the 95:5 HAp:Ag sheet. Further reduction of viable *S. aureus* with increased dead bacterial cells was observed on the 75:25 HAp:Ag. 

We then quantified the viable bacterial colonies to confirm the results acquired from the biofilm confocal imaging. In addition to quantifying the bacteria that attached and grew on the material, we also quantified the non-adherent, planktonic bacteria that were still able to form viable colonies. As expected, the non-adherent bacteria in the media were viable in the absence of an anti-infective agent (uncoated PMMA and nHAp-coated PMMA) (Figure 5B). In contrast, the bacteria in suspension were largely eliminated with the addition of nAg in the coating layer. All three substrates, 99:1 HAp:Ag, 95:5 HAp:Ag, and 75:25 HAp:Ag, achieved >99% of killing efficiency against both adherent and non-adherent *P. aeruginosa*, when compared to the groups without Ag addition, PMMA, and nHAP (Figure 5B). 

Consistent with the confocal imaging, both non-adherent and adherent *S. aureus* were viable without the presence of Ag (Figure 5C). The addition of a low concentration of nAg [0.2% (*w*/*v*)] into the nHAp-coated PMMA was not able to inhibit *S. aureus* growth. Only with 95:5 HAp:Ag and 75:25 HAp:Ag substrates, a >99% killing efficiency against non-adherent and adherent *S. aureus* was achieved.

### 2.3. Biocompatibility of Dual-functionalized PMMA Substrates

**Figure 6:**
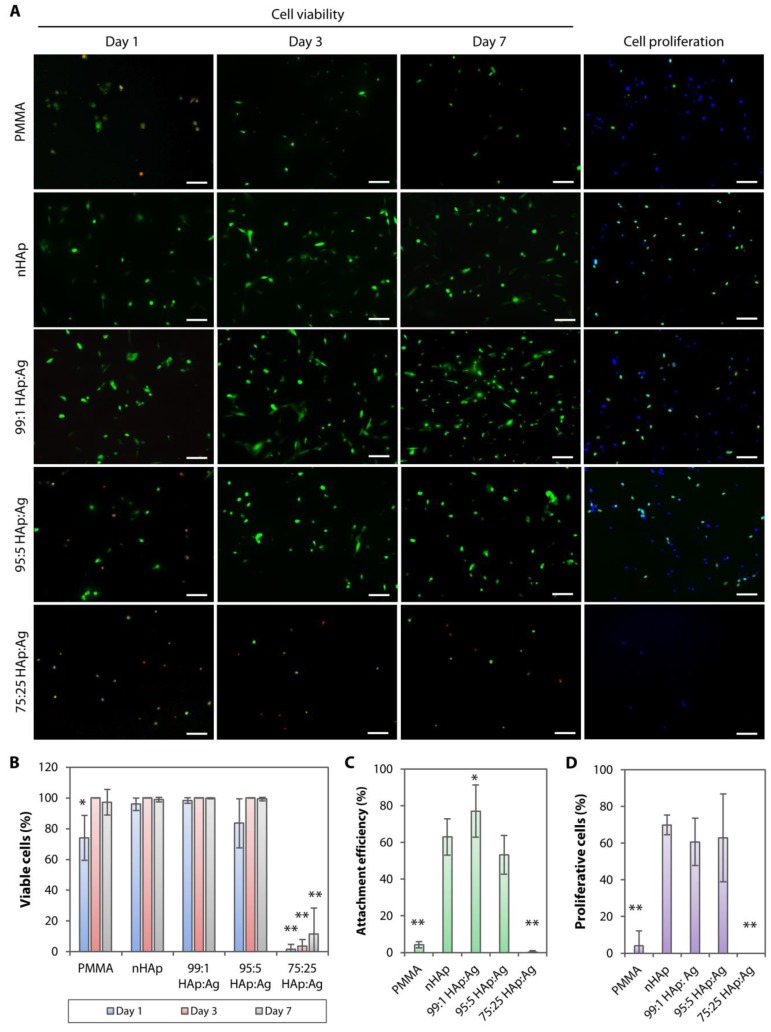
Cell viability, attachment efficiency, and proliferation rate on hydroxyapatite and silver-coated PMMA sheets. (**A**) Live (green)/dead (red) cell assay showing stromal fibroblast viability on days 1, 3, and 7 of cell culture. Cell proliferation analysis was performed on cells that had been cultured for 48 h. Proliferative cells (EdU-positive cells) are stained in green. 4’,6-diamidino-2-phenylindole (DAPI) (blue) was used to stain the cell nuclei. Scale bars = 100 µm. (**B**) Extremely poor cell viability was observed on the 75:25 HAp:Ag substrate. The other two groups, 99:1 HAp:Ag and 95:5 HAp:Ag, had comparable cell viability to the nHAp group. The bar graph summarizes the percentage of viable cells after culturing on the untreated, nHAp-coated, and nHAp and Ag-coated PMMA substrates. (**C**) Cell attachment was poor on the untreated PMMA and 75:25 HAp:Ag. The bar graph shows the percentage of attachment efficiency on the untreated and coated PMMA substrates. (**D**) Cell proliferation rate was also poor on the untreated PMMA and 75:25 HAp:Ag. The bar graph shows the cell proliferation rate on the untreated and coated PMMA substrates. In all bar graphs, the height of bars and height of error bars represent the mean value and standard deviation of the mean, respectively. * *p* < 0.05 and ** *p* < 0.001 relative to nHAp group.

Consistent with our previous studies [21,22], corneal stromal fibroblasts seeded on untreated PMMA for 1 day remained non-adherent, and had a poor viability and cell proliferation rate (Figure 6A). It appeared that the cell viability did not reduce further at days 3 and 7 of culture (Figure 6A). The cell viability (Figure 6B), attachment efficiency (Figure 6C), and proliferation rate (Figure 6D) were significantly increased when the cells were cultured on the nHAp substrate (*p* = 0.002, *p* = 9.5 × 10^−8^, and *p* = 1.1 × 10^−11^, respectively). The cells maintained excellent viability over 7 days. Similarly, stromal fibroblasts cultured on 99:1 HAp:Ag had excellent viability over 7 days. There was a slight reduction in cell viability on the 95:5 HAp:Ag on day 1, but it recovered in the following days of culture. The attachment efficiency and proliferation of both groups were comparable to the nHAp group (Figure 6A). The 99:1 HAp:Ag group resulted in the best attachment efficiency (Figure 6C), which was most likely attributed to its superior surface hydrophilicity level (Table 1). In contrast, there were many dead cells observed on the 75:25 HAp:Ag substrate (Figure 6A). The cell viability remained poor over 7 days—1.6 ± 3.1% on day 1, 3.5 ± 4.4% on day 3, and 11.5 ± 16.8% on day 7 of culture (Figure 6B). Naturally, due to the high level of cell death, the cell attachment efficiency and proliferation rate were significantly lower than that of the nHAp group (*p* = 8.8 × 10^−8^ and *p* = 2.0 × 10^−10^, respectively) (Figure 6C,D). 

### 2.4. Longer-Term Performance of Dual-functionalized PMMA Substrate

In addition to assessing the performance stability of the immobilized nAg by cultivating *P. aeruginosa* and *S. aureus* on 95:5 HAp:Ag substrates that had been incubated in artificial tear fluid (ATF), pH 7.4 for 28 days, the experiment model also simulated a late bacterial infection. We chose the 95:5 HAp:Ag for this experiment because this combination showed the best selectivity in terms of good Gram-positive and Gram-negative bactericidal performance and good biocompatibility. The 99:1 HAp:Ag had good biocompatibility, but was not effective against *S. aureus*. On the other hand, the 75:25 HAp:Ag had an excellent *P. aeruginosa* and *S. aureus* killing ability, but was extremely toxic to the corneal stromal cells. 

**Figure 7:**
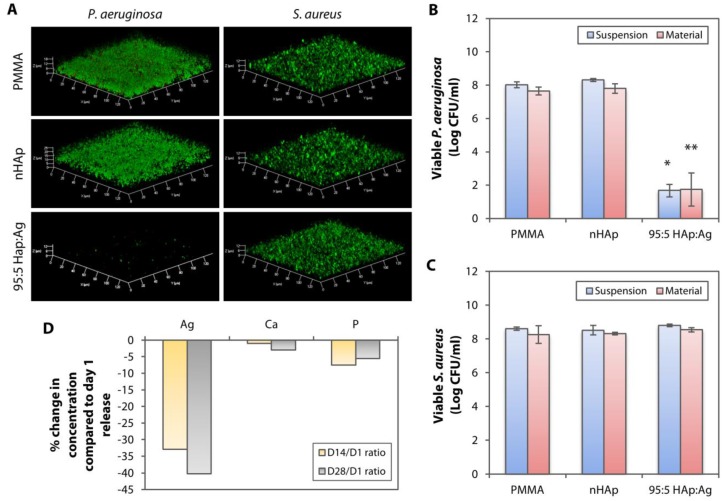
Longer-term performance of dual-functionalized PMMA following a 28-day incubation in an aqueous environment. (**A**) Untreated, nHAp, and 95:5 HAp:Ag substrates were incubated in artificial tear fluid (pH 7.4) for 28 days before being cultivated with *P. aeruginosa* and *S. aureus*. Laser confocal microscopy revealed that the 95:5 HAp:Ag inhibited *P. aeruginosa* biofilm formation, but not the *S. aureus* biofilm. (**B**) Bacterial quantification showing efficient growth inhibition (>99% killing efficiency compared to the PMMA and nHAp groups) of both non-adherent *P. aeruginosa* (labeled as suspension) and *P. aeruginosa* that was still attached on the substrates (labeled as material) with the dual-functionalized PMMA. The bar graph summarizes the viable CFU of *P. aeruginosa* in the presence of untreated, nHAp-coated, and nHAp, and Ag-coated PMMA substrates. (**C**) In contrast, the 95:5 HAp:Ag did not inhibit the non-adherent and adherent *S. aureus*. The bar graph summarizes the viable CFU of *S. aureus* in the presence of the untreated and coated PMMA substrates. * *p* < 0.05 and ** *p* < 0.05 relative to viable bacteria in suspension and attached on material, respectively, in the nHAp group. (**D**) ICP-MS detected in the concentration of Ag ions in the aqueous environment after 14 and 28 days of incubation, respectively, compared to incubation day 1.

From the biofilm assay and imaging, both strains of bacteria were able to form biofilms on the untreated and nHAp-coated PMMA (Figure 7A). The 95:5 HAp:Ag was effective in preventing *P. aeruginosa* biofilm formation, but did not prevent *S. aureus* biofilm growth (Figure 7A). We confirmed the confocal observation with viable bacterial quantification on Tryptic Soy Agar (TSA) plates. The 95:5 HAp:Ag substrate achieved >99% *P. aeruginosa* killing efficiency, regardless of adherent or non-adherent bacteria (Figure 7B). In contrast, the same substrate did not inhibit both non-adherent and adherent *S. aureus* growth (Figure 7C). The viable bacterial count was comparable to the non-infective PMMA and nHAp substrates.

The likely explanation of the reduced bactericidal efficiency of the 95:5 HAp:Ag could be the reduction of Ag^+^ concentration that was released from the nAg residing on the PMMA surface. ICP-MS detected progressively lower amount of Ag^+^ in the ATF, after 14 and 28 days of material incubation. We detected 0.057 ± 0.013 µg/mL and 0.051 ± 0.020 µg/mL of Ag^+^ after 14 and 28 days of incubation, respectively. These values were approximately 33% and 40% reduction from Ag^+^ that was released on day 1 (Figure 7D). The Ca and P concentrations were also reduced compared to the solution collected after 1 day of incubation in the ATF, but the changes were not as pronounced as the Ag (Figure 7D).

## 3. Discussion

Medical devices have played an essential role in improving and advancing the healthcare system, including the use of KPros in providing vision to patients with severe corneal diseases and also to patients that suffer from multiple donor graft rejection [25]. However, cases of antibiotic-resistant bacterial infections, which are normally caused by the attachment of bacteria and subsequent formation of biofilm on the medical devices, have been a critical issue for clinicians and patients [26]. Therefore, it is crucial to find an effective antibacterial coating to prevent biofilm formation on medical devices. Silver is one of the oldest metals that has been utilized as an antimicrobial material [27]. Many studies have used its intrinsic antimicrobial activity by incorporating nAg or salts into soft and biodegradable polymeric coatings that overlay the medical devices [14,15,16,17,28]. We took an alternative approach by immobilizing the nAg directly onto the surface of a hard polymer (PMMA), which was also coated with nHAp, to support the adhesion and growth of corneal stromal cells [22]. We found that the dual-functionalized PMMA with a low concentration of Ag (99:1 HAp:Ag group) was effective in preventing biofilm formation of Gram-negative pathogen, *P. aeruginosa*. While the dual-functionalized PMMA was still able to inhibit the Gram-positive pathogen, *S. aureus*, a higher concentration of Ag in the coating (95:5 HAp:Ag group) was needed to achieve >99% killing efficiency. The high concentration of Ag in the coating (75:25 HAp:Ag group) was effective in killing both pathogens, but was extremely toxic to the fibroblasts. 

The motivation to develop an anti-infective bioactive coating was born from our previous attempt to improve the KPro biointegration with the nHAp coating [22], where, we established a dipcoating method to immobilize nHAp on the surface of KPro PMMA optic. We showed that the coating resulted in a rough and hydrophilic surface, which was beneficial for stromal cell adhesion and proliferation. However, the rough and hydrophilic surface can also render the material more susceptible to bacterial adhesion [29,30,31]. Therefore, the addition of nAg in the bioactive coating on the PMMA optic could potentially prevent bacterial adhesion and subsequent biofilm formation, benefitting the device longevity.

The biological and bactericidal effects of Ag are believed to be largely attributed to the free Ag^+^ that is released from the NPs [12,32]. Ag in the form of either ions or NPs can be toxic to human cells, e.g., human mesenchymal stem cells, peripheral blood mononuclear cells, fetal lung fibroblasts, and hepatocellular carcinoma cell line [33,34,35]. It is difficult to gauge the safe Ag^+^ dosage for human cells, but yet is potent enough to kill bacteria, as it appears that every cell type has a different tolerance to the Ag^+^ toxicity. Recent publications by Greulich et al. [34] and Vukomanovic et al. [35] reported that the toxic concentration of Ag towards bacteria and human cells occurred at almost the same level. Here, we showed that 95:5 HAp:Ag was selective in its killing target. It could be due to the Ag^+^ concentration “sweet spot”, where the concentration was high enough to kill the *S. aureus* and *P. aeruginosa*, but was low enough to not cause toxicity to human stromal fibroblasts. Increasing the amount of Ag in the coating (75:25 HAp:Ag group), however, induced cytotoxicity due to the high concentration of Ag^+^ (0.690 ± 0.167 µg/mL) that was released into the aqueous environment. This amount was 8 times higher than the Ag^+^ released from the 95:5 HAp:Ag (*p* = 0.024). Ag in the form of NPs becomes toxic when internalized by cells, by disrupting protein trafficking, protein denaturation (alpha-beta transition), and local depletion of glutathione and other anti-oxidants [36]. The nAg most likely did not play any role in cytotoxicity, in our case, as they were well-anchored at the surface of the PMMA and unlikely to be delaminated. We showed that in normal (ATF, pH 7.4) and diseased (ATF, pH 5) microenvironments, the coating was not significantly degraded (no changes in the weight of materials over 4 weeks) (Figure S1A,B) and the surface morphology remained unchanged (Figure S1C). 

There are few publications with respect to the threshold of acute Ag toxicity to corneal cells. Most morbidity is associated with chronic exposure to Ag, with the development of irreversible pigmentation of the cornea and conjunctiva, due to metal precipitation, a condition known as argyrosis. The affected eyes typically become bluish gray or ash gray in areas exposed to sunlight [37]. Although the Ag deposits remain in the corneal and conjunctival cells, they usually do not cause any detrimental changes to the cells [37,38]. However, the direct effects of free Ag^+^ on human corneal cells have not been studied extensively. A study by Santoro and colleagues showed that 8 µM of AgNO_3_ was able to kill immortalized corneal epithelial cells (HCE-T cell line), but did not address the Ag^+^ concentration in the culture media [39]. We cannot determine the threshold of Ag^+^ toxicity to corneal stromal fibroblasts based on the study here, but we can extrapolate a range between the safe Ag^+^ level (released by 95:5 HAp:Ag coating) and the toxic Ag^+^ concentration (produced by 75:25 HAp:Ag coating).

Based on the biocompatibility test and bacterial biofilm assay, the 95:5 HAp:Ag coating showed the best selectivity among the three dipcoating formulations that contained nAg. The 99:1 HAp:Ag coating had good biocompatibility and even resulted in a significantly better cell adhesion efficiency, compared to the nHAp (*p* = 0.037), but it was ineffective in preventing *S. aureus* biofilm formation. On the other hand, the 75:25 HAp:Ag coating combination was not suitable for our application in the cornea due to its cytotoxicity. With the 95:5 HAp:Ag coating, we were able to achieve >99% inhibition of *S. aureus* and *P. aeruginosa* growth, when compared to the PMMA and nHAp (groups without Ag); therefore, preventing any biofilm formation. A simulation of late-onset bacterial infection showed that the 95:5 HAp:Ag could prevent *P. aeruginosa* biofilm formation, but not *S. aureus* biofilm growth, up to the duration tested in this study (28 days). We were able to relate this reduced efficiency to the decrease of free Ag^+^ released from the coating to a level that was insufficient to inhibit the *S. aureus*. Previous studies have also noted a lower Ag bactericidal efficiency against *S. aureus,* compared to *P. aeruginosa* [40,41], which was most likely contributed by the thicker peptidoglycan cell wall in *S. aureus* [42].

Medical devices that are composed of materials capable of providing a long-term anti-infective coating against any pathogen have always been desired. Our findings suggest that nAg incorporation or coating on medical devices provides excellent protection against perioperative and early postoperative infections, but is less effective as a long-term solution in preventing bacterial infections, especially in preventing *S. aureus* infection. However, the longer-term sustenance of killing efficacy against *P. aeruginosa* should be recognized, considering the increasing number of reports on multidrug-resistant (MDR) *P. aeruginosa* [43], including cases of MDR *P. aeruginosa* keratitis on the ocular surface [44,45]. We showed that the diminishing bactericidal effects were due to the reduction of Ag^+^ level, over time, in the bodily fluid. This finding is consistent with others, who have also noticed a reduction of Ag^+^ released from nAg that have been incorporated in soft polymers or thermal-sprayed on metal surfaces [46,47]. We would also want to highlight that many studies in the literature typically only assessed bacterial inhibition on substrates that were freshly coated with Ag [14,16,17,30,39,41], but did not take into account the potential reduction of Ag^+^ and the consequent reduction in bacterial killing efficiency in a bodily fluid. Such knowledge of anti-infective efficiency is important to dictate whether the coating should be used to combat perioperative infections or long-term infections and also to determine the intended usage of the to-be-coated target materials.

In summary, we were able to immobilize two different types of NPs (Ag and HAp) on the surface of a hard polymer, PMMA. The coating created a dual-functionalized substrate that was capable of killing bacteria (a function provided by Ag) and corneal stromal fibroblast support (a function provided by HAp). The 95:5 HAp:Ag coating was sufficient in preventing *P. aeruginosa* and *S. aureus* biofilm formation on the surface of the polymer, and was not toxic to the human corneal stromal fibroblasts. In addition, the coating was also effective in eliminating non-adherent, planktonic bacteria. However, Ag^+^ released from surface-immobilized nAg diminishes over time and becomes less effective in suppressing biofilm formation of Gram-positive bacteria, such as *S. aureus*. Our findings suggested that the nAg coating is more suited to be utilized as a protection against perioperative and early postoperative infections rather than as a long-term preventive solution.

## 4. Materials and Methods

The experimental setup is summarized in a schematic diagram in Figure 2.

### 4.1. Immobilization of Nanoparticles on Polymer’s Surface 

Immobilization of HAp and nAg on PMMA surface was performed by a dipcoating method as previously described [22]. PMMA sheets, purchased from Goodfellow (Huntingdon, England), were cut into smaller pieces with a dimension of 1 cm × 1 cm × 0.5 mm. Before dipcoating, the sheets were washed in 70% ethanol for 15 min, rinsed extensively with distilled water, and dried in 37 °C incubator. In order to find the most optimized formulation for an antibacterial and non-cytotoxic coating on the PMMA, several combinations of dipcoating solution mixture containing rod-shaped, 60-nm nHAp (MKnano, Missisauga, Ontario, Canada) and spherical 90-nm nAg in 5% (*w*/*v*) PMMA (MW 120,000; Sigma-Aldrich, St. Louis, MO, USA) were tested: (1) 20% (*w*/*v*) nHAp (henceforth, referred to as nHAp group); (2) 15% (*w*/*v*) nHAp and 5% (*w*/*v*) nAg (75:25 HAp:Ag); (3) 19% (*w*/*v*) nHAp and 1% (*w*/*v*) nAg (95:5 HAp:Ag); and (4) 19.8% (*w*/*v*) nHAp and 0.2% (*w*/*v*) nAg (99:1 HAp:Ag). The dipcoating mixture was probe sonicated for 5 min at a 50% amplification level with a 5-s pause every 5 s. The dipcoating was then carried out with an automated KSV NIMA dip coater (Biolin Scientific, Stockholm, Sweden). Once the PMMA sheet was clamped on the apparatus, it was dipped for 60 s. The PMMA was lowered to and withdrawn from the dipcoating solution with a speed of 240 mm/min. Following this, the coated PMMA sheet was air-dried for 30 min and dried in a 37 °C incubator, overnight. 

After complete drying, the surface of the coated substrates was subjected to oxygen plasma treatment to remove contaminants from the dipcoating process. Plasma treatment was performed in a Covance multi-purpose plasma system (Femto Science, Seoul, South Korea) with radio frequency oxygen plasma of 200 W power, for 5 min. The PMMA sheets were placed between two parallel plate electrodes enclosed in a plasma reactor chamber. Air was removed with vacuum application for at least 30 min before the power was turned on. The pressure at the moment of plasma discharge was 0.2 Torr. The flow rate of oxygen was set at 20 cm^3^/min. After the discharge stopped, the plasma-treated PMMA sheets were removed from the plasma chamber, washed with 70% ethanol and copious amounts of distilled water, and dried in a 37 °C incubator, overnight, before being used for further experiments. Untreated PMMA was used as a control.

### 4.2. Analysis of Surface Morphology and Elemental Composition

Surface roughness of PMMA sheets (n = 6 in each group) was analyzed using a Nanoscope IIIa atomic force microscope (AFM; Digital Instruments, Santa Barbara, CA, USA). Topographic images were captured in tapping mode, employing monolithic silicon NCH-50 Point Probe (NanoWorld AG, Neuchatel, Switzerland). RMS value was averaged from 3 different scan areas from each sample. Surface morphology of PMMA sheets was observed by SEM. In brief, the PMMA sheets were mounted on a specimen stub secured by carbon adhesive tape. The sheets were sputter-coated with a 10-nm-thick layer of platinum and then examined with a JSM-7600F microscope (JEOL, Tokyo, Japan). Surface elemental composition was assessed by EDX spectroscope attached to the SEM. Percentage of an elemental concentration was averaged from 3 random scan areas of each sample.

### 4.3. Analysis of Water Contact Angle

WCA or level of hydrophilicity of the samples (n = 6 in each group) was measured by a DataPhysics Instruments OCA 15EC contact angle goniometer (Filderstadt, Germany), using a static sessile drop method. WCA of each sample was measured once. At room temperature, distilled water was pumped out of a syringe at a volume of 5 µL. The water was then allowed to settle on the samples for 10 s, before the image was taken.

### 4.4. Analysis of Trace Elements in Aqueous Solutions

Presence of Ag, Ca, and P ions due to material leaching following 1-day incubation in an aqueous environment was detected using an Agilent ICP-MS apparatus (Agilent Technologies, Santa Clara, CA, USA). Coated PMMA sheets (n = 3 in each group) were incubated in 5 mL of distilled water for 24 h, in a 37 °C incubator. Samples that were used for longer-term assessments were incubated in ATF, pH 7.4. The ATF was prepared as previously described by mixing 0.67% (*w*/*v*) of NaCl (Sigma-Aldrich), 0.22% (*w*/*v*) of NaHCO_3_ (Sigma-Aldrich), 0.008% (*w*/*v*) of CaCl_2_·2H_2_O (Sigma-Aldrich), and 0.14% (*w*/*v*) of KCl (Sigma-Aldrich) in distilled water [48]. The ATF was refreshed daily. The ATF was substituted with distilled water, 24 h before the pre-determined collection timepoints (days 14 and 28). The substitution was to eliminate the detection of Ca and P elements present in the ATF, through the ICP-MS, in the final analysis. The following parameters of ICP-MS were applied to assess the trace elements in water—1500W radio frequency (RF) power; 1.8V RF matching; 10 mm sampling depth; 0.2 mm torch-H; −0.6 mm torch-V; 1.07 L/min gas flow rate; 0.1 rps nebulizer pump; and 2 °C spray chamber (S/C) temperature. 

### 4.5. Bacterial Biofilm Assay and Imaging

PMMA samples (n = 3 in each group) were placed in 8-well Ibidi µ-slides (Martinsried, Germany). *S. aureus* (ATCC 15981-gfp) or *P. aeruginosa* (PAO1-gfp) were gifted by Prof. Michael Givskov (Singapore Centre for Environmental Life Sciences Engineering). They had a starting inoculum of 0.5 McFarland Standard (1.5 × 10^8^ CFU/mL) in 200 µL of medium and were cultivated in the presence of the PMMA samples for 3 days to form the biofilms. The bacteria were tagged with green fluorescent protein (GFP), which emitted green fluorescence when alive. Cultivation of *S. aureus* was carried out in a Tryptic Soy Broth (TSB) medium (BD Biosciences, Franklin Lakes, NJ, USA) at 37 °C. Meanwhile, *P. aeruginosa* was maintained in an ABT minimal medium (AB medium containing 2.5 mg/L thiamine) (Sigma-Aldrich), supplemented with 2 mg/mL glucose (Sigma-Aldrich) and 2 mg/mL casamino acids (Sigma-Aldrich) at 37 °C. The cultivation medium was refreshed every 24 h. Propidium iodide was used to stain the dead bacteria. Imaging of biofilms was performed using a LSM780 confocal laser scanning microscope (Carl Zeiss, Oberkochen, Germany) with a 63x objective lens, using an argon laser (488 nm excitation, 535 nm emission) for the green fluorescent protein (GFP) observation and a helium laser (535 nm excitation, 617 nm emission) for the red fluorescence emitted by propidium iodide. 

In addition, some PMMA samples were first incubated in ATF, pH 7.4 for 28 days, at 37 °C. The ATF was refreshed daily. After rinsing the PMMA sheets with copious amounts of distilled water and drying, *S. aureus* and *P. aeruginosa* biofilms were grown on the substrates and imaged as described in the preceding paragraph.

### 4.6. Quantification of Viable Bacterial Colonies

*S. aureus* (ATCC 29213) or *P. aeruginosa* (PAO1) with a 0.5 McFarland standard (1.5 × 10^8^ CFU/mL) starting concentration in 200 µL of medium was cultivated on separate PMMA samples (n = 3 in each group) from those used in the biofilm imaging, described in the previous sub-section. Following a 3-day culture, non-adherent bacteria was first removed from the medium. The remaining bacteria, still attached on the PMMA samples, were lifted by bead beating with 2 mm glass beads for 5 min [49]. A serial dilution of both homogenates (non-adherent and adherent bacteria) was plated on Tryptic Soy Agar (TSA; BD Biosciences) and incubated at 37 °C for 24 h. The number of colonies on each plate was then counted and reported as log CFU. 

### 4.7. Culture and Seeding of Human Corneal Stromal Fibroblasts

Research grade cadaveric human corneal tissues were purchased from Lions Eye Institute for Transplant and Research (Tampa, FL, USA). They were preserved in Optisol-GS (Bausch&Lomb Surgical, Irvine, CA, USA) and transported to the laboratory, at 4 °C. The central button was trephined and treated with 20 mg/mL of dispase II (Roche, Basel, Switzerland), followed by gentle scraping to remove corneal epithelium and endothelium. The stromal tissue was digested with 1 mg/mL of collagenase I (Worthington, Lakewood, NJ, USA) in DMEM/F12 (Life Technologies, Carlsbad, CA, USA) for 12 h at 37 °C. Isolated cells were cultured in DMEM/F12 containing 10% fetal bovine serum (FBS; Life Technologies) and 1% antibiotic/antimycotic (penicillin, streptomycin sulfate and amphotericin B; Life Technologies) to generate stromal fibroblasts. At passage 3 to 5, the fibroblasts were seeded at a 10,000 cells/cm^2^ starting density, on UV-sterilized PMMA sheets in 1 mL of culture medium, and cultured for 1, 2, 3, or 7 days, before further tests. 

### 4.8. Cell Viability Assay

The cytotoxicity was analyzed using the Live/Dead^®^ Viability/Cytotoxicity assay kit (Life Technologies) according to the manufacturer’s protocol. In brief, the cells on the PMMA sheets (n = 3 in each group) were incubated with calcein AM and ethidium homodimer-1 (EthD-1), for 45 min, and then washed and mounted in Fluoroshield (Santa Cruz Biotechnology, Santa Cruz, CA, USA). Samples were viewed using a Zeiss AxioImager Z1 fluorescence microscope (Carl Zeiss, Oberkochen, Germany). Live cells were stained green by calcein AM and dead cells were stained red by EthD-1. Both live and dead cells were quantified from 3 random fields of each sample, and the live/dead cell ratio was calculated. Live and dead cells were counted manually with the assistance of ImageJ cell counter to avoid double counting of cells. Cells, which were double-stained with green and red dyes, were considered as dead cells. In addition, cell attachment efficiency after 1 day of culture was calculated using the following formula:

$$\begin{array}{l} Cell\;attachment\;efficiency\;\left(\%\right)\\ \;=\;\frac{Actual\;number\;of\;cells\;in\;a\;viewing\;field}{Theoretical\;number\;of\;cells\;expected\;to\;attach\;in\;a\;viewing\;field}\;\times \;100\% \end{array}$$

where the theoretical number of cells expected to attach in a viewing field of 0.9 × 0.7 mm dimension was 63 with a seeding density of 10,000 cells/cm^2^.

### 4.9. Cell Proliferation Assay

Proliferation of stromal fibroblasts on the PMMA surfaces was assessed using a 5-ethynyl-2’-deoxyuridine (EdU) assay kit (Life Technologies), according to the manufacturer’s protocol. In brief, the cells were incubated in an EdU-containing (10 µM) medium for 48 h. They were then washed with 0.01M PBS, fixed with 4% paraformaldehyde (Sigma-Aldrich), followed by blocking and permeabilization in 0.1% Triton X-100 (Sigma-Aldrich) in 3% bovine serum albumin (Sigma-Aldrich), at room temperature. Incorporated EdU was detected by Alexa Fluor 488 fluorescent-azide coupling Click-iT reaction. Finally, the samples were mounted in Fluoroshield containing 4’,6-diamidino-2-phenylindole (DAPI; Santa Cruz Biotechnology) and viewed under Zeiss AxioImager Z1 fluorescence microscope (Carl Zeiss). 

### 4.10. Statistical Analysis

Data were expressed as mean ± standard deviation. Statistical significance between groups was calculated by one-way ANOVA and post hoc Tukey comparison test. A value of *p* < 0.05 was considered to be statistically significant. All statistical analysis was performed using the SPSS software (version 17.0, SPSS Inc., Chicago, IL, USA).

## Figures and Tables

**Table 1:** Hydrophilicity of pristine and coated PMMA sheets.

	Water Contact Angle	*p* value compared to PMMA	*p* value compared to nHAp
PMMA	68.1 ± 1.1°		2.40 × 10^−9^
nHAp	18.7 ± 2.7°	2.40 × 10^−9^	
99:1 HAp:Ag	17.7 ± 2.7°	2.43 × 10^−9^	0.501
95:5 HAp:Ag	19.2 ± 2.4°	5.61 × 10^−10^	0.752
75:25 HAp:Ag	20.2 ± 2.3°	3.44 × 10^−10^	0.333

## Supplementary Materials

The following are available online at https://www.mdpi.com/2076-0817/8/3/93/s1, Figure S1: Degradation and delamination of nAg and nHAp coating on PMMA sheets. (A) No significant degradation (weight loss) was found in all groups over a 28-day incubation in artificial tear fluid (ATF), pH 7.4 in 37 °C. The ATF was refreshed daily. (B) No significant materials’ weight loss was also noticed over 28 days in ATF, pH 5 in 37 °C. The incubation in acidic ATF simulated a harsh diseased corneal microenvironment that is typically corrosive in nature. (C) Low magnification (500×) and high magnification (30,000×) SEM images of differentially coated PMMA sheets revealed no coating delamination following 28 days of incubation in either ATF, pH 7.4 or acidic ATF. We did not find any area of bare surface that could indicate a sign of coating delamination.
