# Supplementary material for: Surface Immobilization of Nano-Silver on Polymeric Medical Devices to Prevent Bacterial Biofilm Formation
Source: Pathogens. 2019 Jun 28;8(3):93. doi: 10.3390/pathogens8030093 (PMC6789847; doi:10.3390/pathogens8030093)
Supplement: Supplementary file 1 [file pathogens-08-00093-s001.pdf]

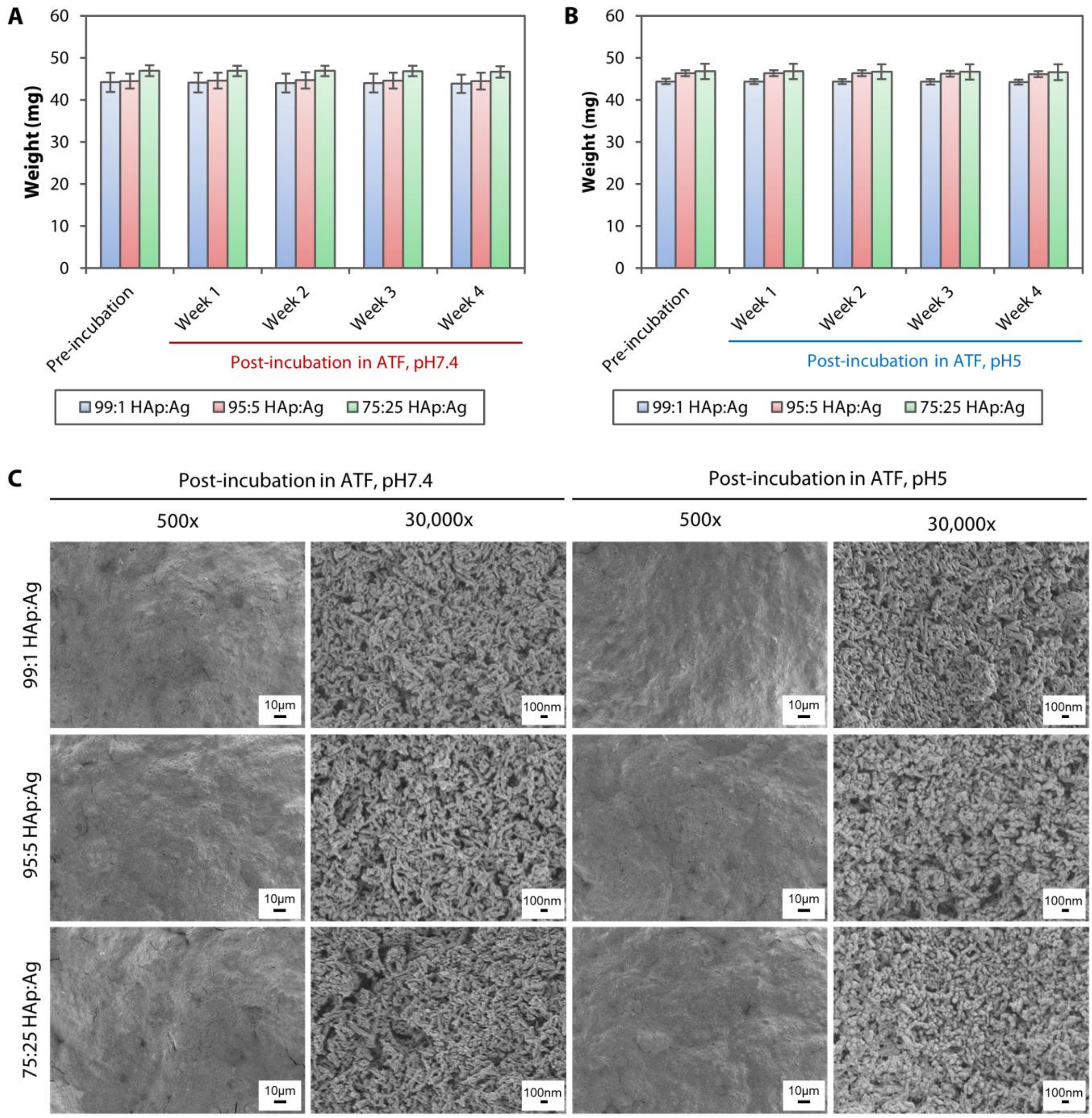


**Figure S1.** Degradation and delamination of nAg and nHAp coating on PMMA sheets. (A) No significant degradation (weight loss) was found in all groups over a 28-day incubation in artificial tear fluid (ATF), pH7.4 in 37°C. The ATF was refreshed daily. (B) No significant materials' weight loss was also noticed over 28 days in ATF, pH5 in 37°C. The incubation in acidic ATF simulated a harsh diseased corneal microenvironment that is typically corrosive in nature. (C) Low magnification (500x) and high magnification (30,000x) SEM images of differentially coated PMMA sheets revealed no coating delamination following 28 days of incubation in either ATF, pH7.4 or acidic ATF. We did not find any area of bare surface that could indicate a sign of coating delamination.
